# Malignant peripheral nerve sheath tumor of the right forearm: Case report

**DOI:** 10.1016/j.ijscr.2025.111800

**Published:** 2025-08-13

**Authors:** Angel Puente Sanchez, Elias Gallardo-Navarro, Brenda Jiménez López

**Affiliations:** a Plastic and Reconstructive Surgery, Instituto Nacional de Pediatría, Mexico City, Mexico; b Resident of General Surgery, Hospital Español, Mexico City, Mexico; c Plastic and Reconstructive Surgery, Hospital Infantil de México, Federico Gómez, Mexico City, Mexico

**Keywords:** Bloc surgical resection, Malignant peripheral nerve sheath tumor, Surgical reconstruction, Surgical skin grafting, Tumor in children

## Abstract

**Introduction and importance:**

Malignant peripheral nerve sheath tumor (MPNST), also called malignant schwannoma, neurofibrosarcoma and neurogenic sarcoma, is a malignant neoplastic lesion originating in the Schwann cells of the sheath lining of peripheral nerves.

**Case presentation:**

A 2-year-old, 7-month-old female patient, was admitted to our clinic due to progressive enlargement of the right forearm, physical examination revealed a tumor on the right forearm, painless, fixed to adjacent structures, hard, with erythema and swelling in the upper part of the lesion, complete surgical excision of the tumor with left inguinal graft was performed, covering the entire resected surgical defect. The pathology report was a malignant peripheral nerve sheath tumor. The patient was discharged with good clinical evolution, the MPNST was low grade so no adjuvant treatment based on radiotherapy or chemotherapy was administered.

**Clinical discussion:**

These malignant tumors are recognized for being aggressive, for high rates of local recurrence and distant metastases, constitute approximately 5 % to 10 % of all soft tissue sarcomas, and arise mainly from peripheral nerve sheath components, with poor prognosis.

**Conclusion:**

This case shows the clinical manifestations and complications that can be expected with these tumors, as well as their reconstructive treatment with adequate esthetic and functional results in young patients.

## Highlights

• A very rare case reported in world literature.
• Research on the subject based on current books and publications
• Adequate treatment options for this pathology.
• Adequate differential diagnoses
• A rare case of an aggressive tumor, with immediate reconstruction after surgical resection, without the need for adjuvant therapy.

## 1 Introduction

Malignant peripheral nerve sheath tumor (MPNST), also called malignant schwannoma, neurofibrosarcoma and neurogenic sarcoma, is a malignant neoplastic lesion originating in the schwann cells of the sheath lining of peripheral nerves, these malignant tumors are recognized for being aggressive, for high rates of local recurrence and distant metastases, constitute approximately 5 % to 10 % of all soft tissue sarcomas, and arise mainly from peripheral nerve sheath components, with poor prognosis [1]. These tumors can develop either de novo or from preexisting nerve sheath tumors, such as neurofibromas, particularly in patients diagnosed with neurofibromatosis type 1 (NF-1) disorder [2]. MPNSTs are associated with syndromes such as NF1, while other tumors are radiation-related or sporadic, the aggressive nature of these tumors is the low survival of affected patients [3]. A factor related to this type of tumor is that sometimes they develop from pre-existing plexiform neurofibromas being more than half of the cases, and sporadic appearances that are not related to NF1, represent about 40 % of cases, and 10 % originate in areas previously exposed to radiation. [3,4]. Differential diagnoses for these tumors include desmoplastic melanoma, cellular schwannoma, atypical neurofibroma, synovial sarcoma, clear cell sarcoma of soft tissue, solitary fibrous tumor, rhabdomyosarcoma, leiomyosarcoma, nodular fasciitis. A history of malignant spindle cell sarcoma arising from the anatomic compartment of a major nerve or having continuity with a neurofibroma or developing in a patient who has NF1, it is straightforward for the pathologist to diagnose an MPNST [4,5]. The anatomical region where these tumors are most frequently located includes the proximal portions of the upper and lower extremities and the trunk [5]. The main therapy for this disease, which is not metastatic, is surgery with adequate oncologic margins, however, it is not always possible due to the location of these tumors and the vascular or nerve structures involved, the size of the tumor is another important factor, resection with disease-free margins sometimes increases patient morbidity, due to the disability or deformity that may occur [6]. The rapid growth of these tumors, accompanied by pain and compressive symptoms, are the main clinical features, in addition to being a noticeable mass growing somewhere in the body [7]. We discuss the case of our patient who was successfully treated with extensive resection, an autologous graft reconstruction, who had no need for neoadjuvant therapy with no local recurrence to date. The work was reported in accordance with 2025 SCARE criteria [8].

## 2 Case report

**Fig. 1:**
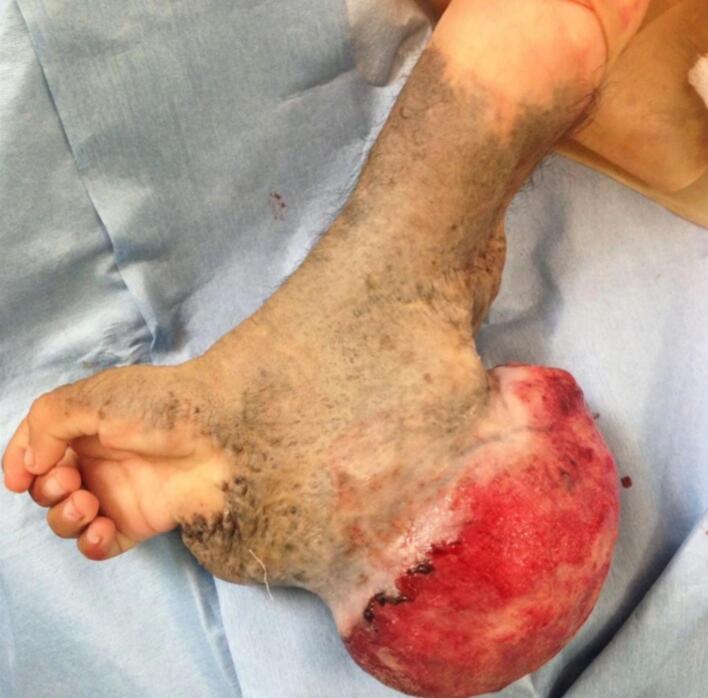
There is a tumor on the right forearm, which on palpation is hard, fixed to adjacent structures, with brown epidermis with 10 × 10 cm nodular ulcerated lesions.

**Fig. 2:**
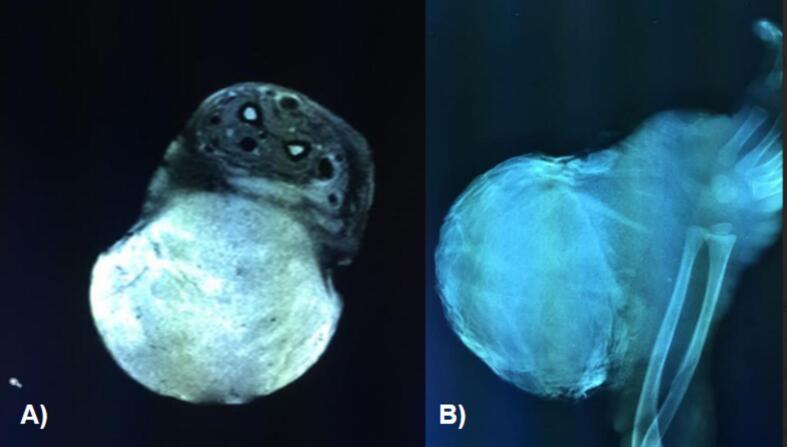
An axial section of a simple computed tomography of the massive heterogeneous tumor measuring 19 × 13 × 10 cm (A), no bone tissue involvement is observed, (B) plain radiography, lateral projection of the right upper extremity, tumor is observed in the right forearm, does not involve the humerus or radius, increased volume of soft tissues.

**Fig. 3:**
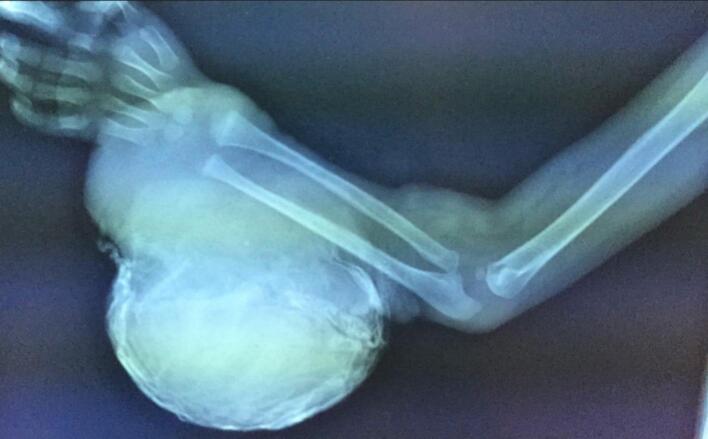
Plain radiography, lateral projection of the right upper extremity, tumor is observed in the right forearm, does not involve the humerus or radius, increased volume of soft tissues.

**Fig. 4:**
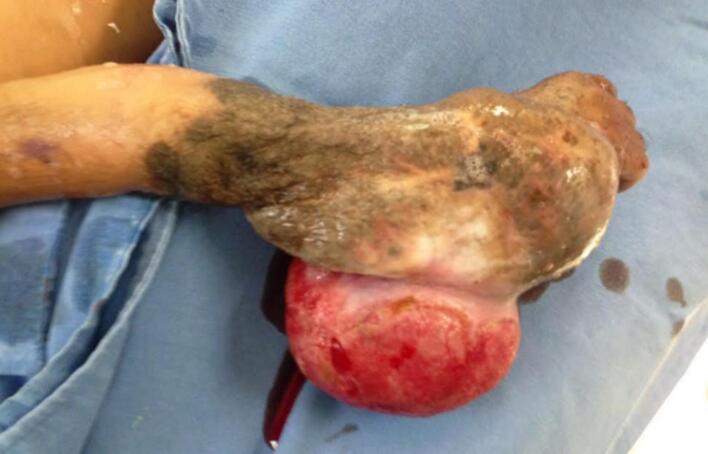
Tumor of 19 × 13 × 10 cm, with brown epidermis with nodular lesion of 10 × 10 cm ulcerated, the lesion is white, yellow homogeneous, well demarcated, covering the dermis and subcutaneous cellular tissue.

**Fig. 5:**
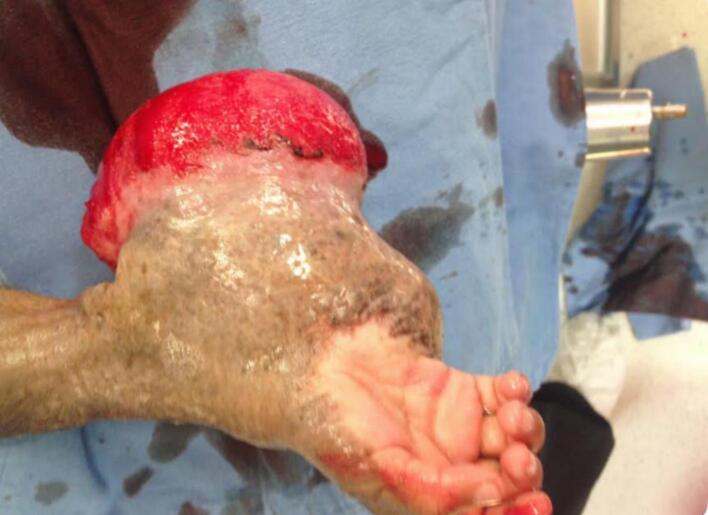
Giant melanocytic nevus is observed covering the entire tumor, palm of the hand free as well as the fingers.

**Fig. 6:**
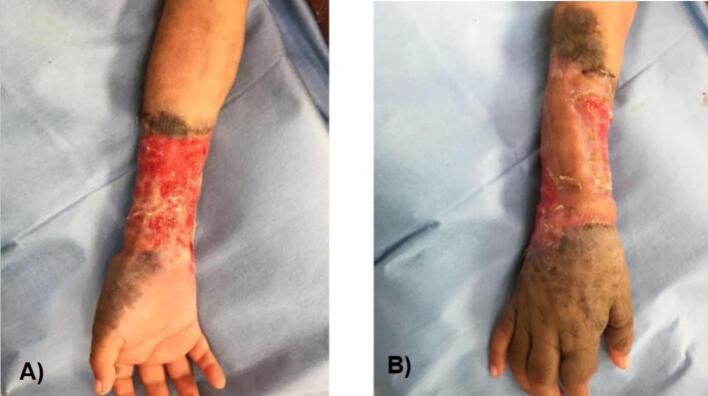
Skin graft of the inguinal region, with adequate coverage of the surgical bed, no tension of the lesion is observed, with adequate coloration and vascular integrity.

**Fig. 7:**
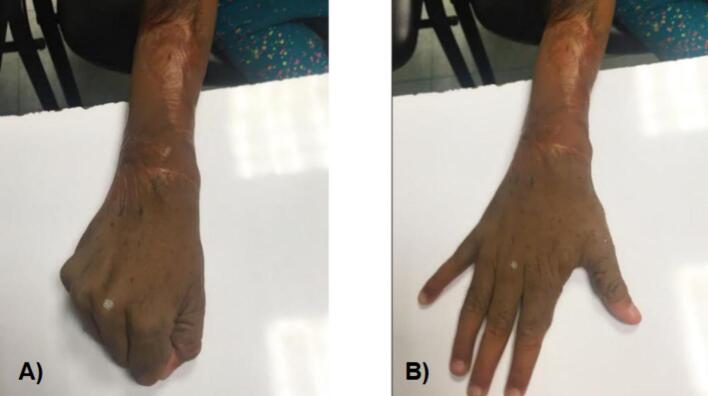
Adequate healing of the graft is observed, with absence of hair, very few changes in color, presence of scar of the surgical procedure, no limitation of movement or pain.

A 2-year-old, 7-month-old female patient, has a history of giant melanocytic nevus since birth, was admitted to our clinic due to progressive enlargement of the right forearm during the last 6 months approximately (Fig. 1). Physical examination revealed a tumor on the right forearm, painless, fixed to adjacent structures, hard, with erythema and swelling in the upper part of the lesion. Routine laboratories were requested, which showed no alterations, the tomography of the extremity of the lesion is observed. An axial section of a simple computed tomography of the massive heterogeneous tumor measuring 19 × 13 × 10 cm (Fig. 2A), no bone tissue involvement is observed, radiographic image (Figs. 2B–3) lateral projection of the right upper extremity, tumor is observed in the right forearm, does not involve the humerus or radius, increased volume of soft tissues, without bone co-involvement. The patient was prepared for complete surgical excision of the mass en bloc (Fig. 4, Fig. 5), a partial thickness autologous graft from the anterior aspect of the left inguinal region of approximately 20 × 15 cm was used, which adequately filled the surgical resection defect, covering the entire resected bed, the bony and vascular structures were preserved, the medial, lateral, superior and inferior borders were free of cancer cell infiltration (Fig. 6). The pathology report was a malignant tumor of the peripheral nerve vein and deep congenital intradermal melanocytic intradermal nevus. The microscopic findings of the specimen are papillary dermis, showing irregularly distributed melanocyte nests, extending focally to the subcutaneous cellular tissue, the nests are separated by dense connective tissue and dilated vessels with irregular walls. The deep dermis and subcutaneous cellular tissue show histologically malignant neoplasm composed of spindle cells with pleomorphic, hyperchromatic nuclei, some with multinucleation and presence of atypical mitoses, the cytoplasm is eosinophilic. The patient was discharged with good clinical evolution, the MPNST was of low grade so no adjuvant treatment based on radiotherapy or chemotherapy was given. After a three-year follow-up, there were no signs of tumor recurrence and the patient had adequate esthetic results (Fig. 7).

## 3 Discussion

The World Health Organization (WHO) categorized MPNST as a soft-tissue sarcoma for the first time in 2013, subtypes of epithelioid, malignant triton tumor, and glandular MPNST were also described and prevalence in the general population is one in 100,000, this pathology occurs equally in both sexes [10,15]. Of the cases reported in this article (Table 1), it is observed that the majority presented this tumor, were women, the in older than 30 years, NF1 only occurred in less than half of the cases, as well as the clinical presentation which was the progressive increase in volume, there were differences in terms of the site of presentation, the majority occurred in the neck, The treatment in most cases was based on surgery with chemotherapy and neoadjuvant radiotherapy especially in tumors larger than 5 cm, these characteristics are consistent with those reported in the guidelines of the (NCCN), however in our case reported there was no need to add neoadjuvant therapy. These tumors may appear de novo or develop from the malignant transformation of a benign neural neoplasm, generally a plexiform neurofibroma [9,10,11]. Other authors mention that these tumors occur more frequently in young males and the regions that come to be observed is the pelvis or in the distal regions of the femur of the lower extremities, when related to NF1, which is a multisystem disorder, caused by a mutation in the germline in the NF gene on chromosome 17q11. 2 which encodes the neurofibromin protein, the absence of this important regulator in cell reproduction is responsible for the appearance of benign and malignant tumors in people with this mutation, this occurs in approximately one in 3500 people worldwide and shows no predominance of gender or ethnicity [10,11]. The definitive diagnosis is made by pathological confirmation, clinical features or by imaging studies may be suggestive of tumors, but confirmation is by histology, there are specific histological features, such as fascicles with alternating cellularity and marbling, palisade/rosette arrangement and asymmetric spindle cells, however in there are specific pathognomonic histopathological features, so sometimes the diagnosis is difficult [9,12]. Among the immunohistochemical stains used for diagnosis is variable expression demonstrating S100 and/or SOX10, these markers may be decreased or absent compared to other tumor types such as schwannomas, also demonstrate loss of neurofibromin expression and trimethylation of H3K27, the latter, is a key epigenetic marker that is lost in MPNST and has a relatively high specificity for diagnosis, however, close mimickers such as melanoma may also demonstrate loss [13,14]. With respect to immunohistochemical markers, the S100 protein has been identified in 50–90 % of MPNST, being an important marker to associate it to a tumor of peripheral nerve sheath origin, since it is also present in synovial sarcomas, cellular schwannoma and fusiform melanoma, however other sensitive and present markers for MPNST are CD34, CD56 and the protein product of gene 9.5 (PGP 9.5), to differentiate it from synovial sarcoma the epithelial membrane antigen (EMA) and CK7 must be negative, which together have high specificity for synovial sarcoma, as well as the proteins AE1/AE3, CD99, TLE1, frequent in that sarcoma [23,24]. The latest 2022 clinical guidelines from the National Comprehensive Cancer Network (NCCN) discussed the main way of MPNST diagnosis, the primary means of imaging and histological features, add genetic mutation analysis and molecular detection during MPNST pathogenesis, which are the latest methods to diagnose MPNST by applying new molecular targets that will help to diagnose and classify MPNST more accurately [15,16]. As mentioned above, within the clinical characteristics that differentiate this type of malignant tumor, is the rapid growth of a tumor in any part of the body, essentially the diagnostic suspicion is made with the important background of NF1, within the imaging studies that are requested, an x-ray to assess the involvement or destruction of bone, MRI to assess margins and possible involvement with other anatomical structures, The characteristics of these tumors that are observed are soft tissue invasion, tumor heterogeneity, poorly defined margins and surrounding edema, in T1, T1 with gadolinium and T2 sequences, and above all for their multiplanar capabilities, FDG-PET also works by assessing intracellular glucose levels in highly metabolic tumor cells [20,22,25]. The need for surgical reconstruction by the plastic surgery service, helps to reduce the sequelae of a wide resection of any region of the body, especially exposed areas as in our patient, which increases morbidity and also are not aesthetically acceptable to the patient, the use of graft or flaps for reconstruction in oncological procedures, offers a treatment with better prognosis with the patient, offering a resection with free margins and adequate reconstruction. The prognosis of MPNST is determined by factors such as large tumor volume, positive surgical margins, increased Ki-67 proliferation index and localization as in head and neck [18,19]. Due to the high local recurrence rate, wide surgical resection is the treatment of choice, in case of positive margins, surgical resection should be repeated, adjuvant radiotherapy is recommended when tumor resection is incomplete or in case of metastasis, chemotherapy is controversial, but should be considered as neoadjuvant treatment in high grade [16,17,19].

**Table 1:** Complementary surgery and radiation therapy.

Authors	Year	Sex	Age	Tumor site	Size	Clinical presentation	Associated syndrome	Surgical treatment	Reconstruction by plastic surgery	Adjuvant treatment
Khushbu Vaidya, et al.	2024	Female	56 years old	Left foot	>5 cm	Complaint of pricking pain, swelling, and sticky whitish discharge from the blackish-pigmented area.	No	Lisfranc amputation of the left foot	No	No
Pezhman Kharaz, et al.	2024	Male	67 years old	Right side of the neck	>5 cm	Presence of mass in the neck, compressive, without pain	No	Bloc resection with segments of vagus nerve and vascular structures	No	Chemotherapy and radiotherapy
Demirbaş A, et al.	2024	Female	37 years old	Left frontal region of the face	<5 cm	Progressive increase in the size of a painless red lump	No	Wide resection with 2 cm margin	Anterolateral thigh perforator free flap to reconstruct the region.	Radiotherapy
Chiara Eberspacher, et al.	2023	Female	61 years old	Perianal mass	>5 cm	Tender mass with distinct boundaries	No	Complete and precise removal of the tumor was performed by en bloc excision.	No	Brachytherapy.
S. Bartier, et al.	2023	Female	62 years old	Right cheek and hard palate	>5 cm	Hardened submucosal tumefaction in the cheek and hard palate, with loosened teeth but no adjacent skin abnormality.	No	Total maxillectomy with resection of the orbital floor through a lateral rhinotomy.	Maxillary prosthesis	Radiotherapy
Aleksandra Borovika, et al.	2023	Female	62 years old	Mass of the right side of the neck.	<5 cm	Progressive increase in volume and pain	No	Excision of solid mass, preservation of the vagus nerve on the right side.	No	Radiotherapy
Hongcang Wang, et al.	2022	Male	36 years old	First distal phalanx of the left thumb	<5 cm	Progressive increase in the size	No	Removal of the tumor tissue.	No	Chemotherapy and Radiotherapy
Mohamed Azharudeen Jr, et al.	2021	Male	30 years old	Left side of the neck	>5 cm	Welling on the left side of the neck, dysphagia, dyspnea and Horner′s Syndrome	NF1	Bloc resection without any other structure	No	Chemotherapy
Senthilkumar A C, et al.	2019	Female	47 years old	Right side of her pelvis.	>5 cm	Progressive increase in volume and pain	Von Recklinghausen Syndrome	Lower laparotomy, en bloc resection of right lateral pelvic wall mass with nodular pelvic lymph node chains	No	Radiotherapy
Stefanie Williams	2018	Female	21 years old	Left lateral thigh	>5 cm	Progressive increase in volume and pain	NF1	Conservative management and monitoring	No	No
Amit Gupta, et al.	2017	Male	70 years old	Occipital region	>5 cm	Swelling in the occipital region of scalp	NF1	Surgical removal of the mass with 2 distance around the base of the tumor	Repair with transposition flap and donor site was repaired with split skin graft from thigh.	Radiotherapy
Rueda-Arenas E, et al.	2016	Female	2 years and 10 months old	Abdominal mass	>5 cm	Progressive enlargement of the abdomen without pain	No	Resection of 80 % of the tumor due to involvement of the iliac artery, ureter, left gonadal artery and femoral vein.	No	Second surgery and radiotherapy

Currently, research on therapeutic targets for MPNST has developed rapidly and it is likely that in the near future clinical trials will incorporate therapies that inhibit components of the epigenetic machinery; however, clinical trials of therapies vary greatly, probably because they focus on pathways related to NF1 and not on sporadic cases [20]. Angelov reported that the disease-free and overall survival rates, were approximately 64 and 30 % at 5 years, respectively, compared with 72 to 78 % reported in soft tissue sarcomas, the MPNST subgroup has a selectively worse prognosis than soft tissue sarcomas, because of high local recurrence [21,22].

## 4 Conclusion

It is a tumor that rarely occurs in patients without NF1 syndrome. Considering that this malignant neoplasm can mimic any benign tumor, therefore, so care must be taken in the diagnosis and initial treatment, histopathological examinations and immunohistochemical analysis are essential for accurate diagnosis and treatment of these tumors, is always surgical removal and adjuvant radiotherapy and chemotherapy should be individualized to not increase the morbidity and mortality of the patient. It requires treatment with safety margins and close follow-up due to its high recurrence, so this type of surgical resection has become disabling or aesthetically unacceptable, surgical reconstruction is part of the treatment for this type of tumor, especially in young patients.

## Contribution statements

Angel Puente Sanchez- Author contribution: Investigation.

Elías Gallardo Navarro Author contribution: Editor.

Brenda Jimenez Lopez Author contribution: Investigation.

## Consent

Written informed consent was obtained from the patient's legal parents for the publication and accompanying images. A copy of the written consent is available for review by the Editor-in-Chief of this journal upon request.

## Ethical approval

This study is exempt from ethical approval in our institute because it is a case report.

## Sources of funding

All authors report that they have not received any financial support from any organization or individual for the submitted work.

## Funding

The authors declare that they did not receive any type of funding for the completion of the article.

## Declaration of competing interest

The authors declare that they have no conflict of interest.
